# An Empirical Study of Training Data Selection Methods for Ranking-Oriented Cross-Project Defect Prediction

**DOI:** 10.3390/s21227535

**Published:** 2021-11-12

**Authors:** Haoyu Luo, Heng Dai, Weiqiang Peng, Wenhua Hu, Fuyang Li

**Affiliations:** 1 School of Computer Science, South China Normal University, Guangzhou 510631, China; hluo@m.scnu.edu.cn; 2 School of Mechanical and Electrical Engineering, Wuhan Qingchuan University, Wuhan 430204, China; daiheng726@163.com; 3 School of Computer Science, Wuhan University, Wuhan 430072, China; pengweiqiang@whu.edu.cn; 4 School of Computer Science and Artificial Intelligence, Wuhan University of Technology, Wuhan 430070, China

**Keywords:** fault prediction, machine learning, data selection

## Abstract

Ranking-oriented cross-project defect prediction (ROCPDP), which ranks software modules of a new target industrial project based on the predicted defect number or density, has been suggested in the literature. A major concern of ROCPDP is the distribution difference between the source project (aka. within-project) data and target project (aka. cross-project) data, which evidently degrades prediction performance. To investigate the impacts of training data selection methods on the performances of ROCPDP models, we examined the practical effects of nine training data selection methods, including a global filter, which does not filter out any cross-project data. Additionally, the prediction performances of ROCPDP models trained on the filtered cross-project data using the training data selection methods were compared with those of ranking-oriented within-project defect prediction (ROWPDP) models trained on sufficient and limited within-project data. Eleven available defect datasets from the industrial projects were considered and evaluated using two ranking performance measures, i.e., FPA and Norm(Popt). The results showed no statistically significant differences among these nine training data selection methods in terms of FPA and Norm(Popt). The performances of ROCPDP models trained on filtered cross-project data were not comparable with those of ROWPDP models trained on sufficient historical within-project data. However, ROCPDP models trained on filtered cross-project data achieved better performance values than ROWPDP models trained on limited historical within-project data. Therefore, we recommended that software quality teams exploit other project datasets to perform ROCPDP when there is no or limited within-project data.

## 1. Introduction

Software defect prediction (SDP), also known as software fault prediction, is a research hotspot, which has drawn lots of attention from both industry and academia [1,2]. Defect prediction recognizes the appearance of defects in the system or industrial software, which provides support to find the category, location, and scale of defects [3,4,5,6,7]. It has long been recognized as one of the important aspects of improving the reliability of industrial system software [8,9,10]. With the development of artificial intelligence algorithms, the reliability of automatic defect prediction is ever-increasing. The general method of software defect prediction models is to learn a classification model from the historical datasets via the machine learning algorithms, and then predict whether new software modules contain bugs [11]. The accurate prediction results can contribute to the allocation of reasonable testing resources by focusing on those predicted defect-prone modules [12,13].

Within-project defect prediction (WPDP) models perform well when sufficient historical within-project (WP) data are available to construct a SDP model [14,15]. However, it is very hard for a new project to construct a WPDP model with limited historical data. Cross-project defect prediction (CPDP) is an efficient approach proposed and adopted by the research community to alleviate the problem. CPDP techniques firstly use the other project datasets (i.e., CP data) to construct a SDP model, and then predict the defect-proneness of WP software modules [16]. However, only predicting the defect-proneness of the WP modules is not very efficient for limited resource allocation, because the CPDP model will allocate equal testing resource to all predicted defect-prone modules, without considering the number of defects or defect density of the modules [17,18]. Ranking-oriented cross-project defect prediction (ROCPDP) can rank these WP modules according to their predicted defect numbers or densities, thereby allocating the limited testing resources more efficiently.

Turhan et al. [19] found that using all available CP data for model construction results in a CPDP model with low performance due to existence of several irrelevant CP modules within the data. Therefore, they proposed a training data selection method (i.e., nearest neighbor filtering) to filter out irrelevant CP modules. Their experimental results demonstrated that the filtered data based on the training data selection method do improve the performances of CPDP models, but it is a challenge for CPDP models to outperform WPDP ones. Inspired by this work [19], many novel training data selection methods have been proposed to find the most relevant training data for CPDP [20,21,22,23,24,25]. These studies demonstrated the benefits of training data selection methods on CPDP performance, but the practical benefits of these methods in urgent defective modules (i.e., software modules with more defects or higher defect density) are unknown. Empirical evidence on the impacts of training data selection methods on the performances of ROCPDP models is thus necessary. Knowing the impacts of these data selection methods on the performances of ROCPDP models could help software quality teams to make effective decisions regarding scarce resource allocations and prioritization of defective modules.

Accordingly, we investigated the practical benefits of applying nine training data selection methods for ROCPDP on eleven available defect datasets and evaluated them using two ranking performance measures, i.e., FPA and Norm(Popt). The experimental results indicate that the data selection methods have no positive impacts on the performance of ROCPDP. Additionally, we investigated the impacts of training data selection methods on the performance of ROCPDP compared to ranking-oriented within-project defect prediction (ROWPDP) models trained on sufficient and limited within-project data. The experiment results indicate that the performances of the ROCPDP models trained on filter CP data are not comparable with those of ROWPDP models trained on sufficient historical WP data in terms of FPA and Norm(Popt); and ROCPDP models trained on filtered CP data achieved better results than ROWPDP models trained on limited historical WP data.

In real life, a new software project may not contain sufficient training data to build a ROWPDP model with high performance. However, there are many historical data from public software repositories. Therefore, we recommend that software testers exploit other project datasets to conduct ROCPDP at the early stages of development activities when there is no or limited WP data.

In summary, this paper makes the following contributions:

(1) We conducted an extensive comparative study on the impacts of nine training data selection methods on the performance of ROCPDP. To the best of our knowledge, this was the first attempt to perform such a large-scale empirical study on training data selection methods in the context of ROCPDP.
(2) We evaluated the performances of training data selection methods using both the module-based ranking performance measure (FPA) and the LOC-based ranking performance measure (Norm(Popt)), and employed a state-of-the-art multiple comparison technique (double Scott–Knott test) to rank and cluster the training data selection methods into distinct groups. Therefore, software quality teams are provided with more choices for practical applications.

The remainder of this paper is organized as follows. Section 2 presents the background and related work. Section 3 briefly introduces the training data selection methods. Section 4 and Section 5 present the experiment setup and experiment results, respectively. Finally, Section 6 presents the conclusion and points out the future work.

## 2. Related Work and Background

We first introduce the motivation behind the ROCPDP study. Then, we introduce the existing SDP and ranking-oriented defect prediction (RODP) methods. Finally, we summarize most existing works on CPDP.

### 2.1. ROCPDP

**Figure 1:**
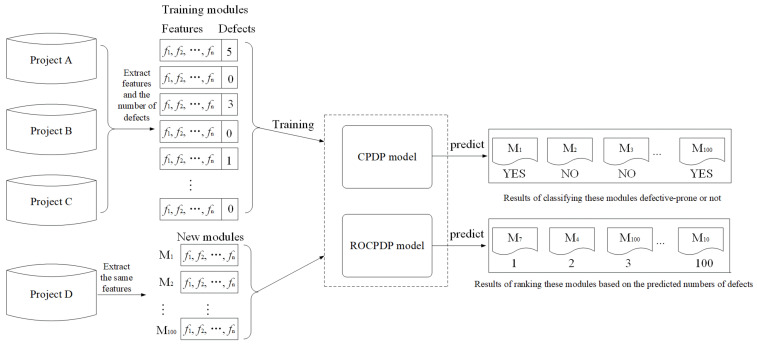
The difference between a CPDP model and a ROCPDP model.

ROCPDP can rank WP modules according to their predicted bug number or density, thereby assigning the testing resources more efficiently. We use an example in Figure 1 to illustrate the difference between CPDP and ROCPDP.

**Example:** Project D is a new developed project which has one hundred software modules. As of the deadline, the software testers could only test a fraction of these modules (e.g., twenty modules). Since Project D is a new project and there are no historical data, the testers first used multiple CP datasets (i.e., Project A, Project B, and Project C) to build a classification model for predicting whether these modules are defective, or a model for ranking the modules according to their predicted bug numbers or densities. Then, they employed the built model to predict the defect-proneness of the modules or rank them. Suppose that the classification model predicts that forty modules are defective: the software testers face a problem as to which twenty modules among the forty predicted defective ones should expect to be tested. The testers could, however, test the first twenty based on the results of the ranking model, i.e., first inspecting the modules with more bugs or higher defect density. Consequently, ranking modules in Project D can be more helpful than predicting whether or not a module is defective in the absence of testing resources. Since this CPDP approach employs the learning to rank technique to build models, we call it ranking-oriented cross-project defect prediction (ROCPDP) [26].

To the best of our knowledge, only one work has focused on the ROCPDP problem. You et al. [27] employed a simple multiple linear regression model to minimize the difference between the ranking list of WP modules and that of CP modules. However, this work overlooked that the existence of larger irrelevant CP modules in CP datasets. Therefore, we proposed the first research question (RQ) to investigate the practical benefits of applying training data selection methods to filter out irrelevant CP modules for ROCPDP: **RQ1: Which training data selection method leads to better performance for ROCPDP?**

In addition, in general, ROWPDP works well if sufficient historical WP data are available to train a defect prediction model. Therefore, we proposed the second research question to compare ROCPDP models trained on filter CP data and ROWPDP models trained on sufficient historical WP data: **RQ2: How does the application of training data selection methods in ROCPDP models perform compared to ROWPDP models trained on sufficient historical WP data?**

However, in practice, it is rare to obtain sufficient training data for a new project. Therefore, software quality teams perform ROWPDP with limited historical WP data, or employ other project datasets to build ROCPDP models. Therefore, we wanted to clarify how well ROWPDP models perform with limited historical WP data, compared with these training data selection methods in the third research question: **RQ3: How does the application of training data selection methods in ROCPDP models perform compared to ROWPDP models trained on limited historical WP data?**

### 2.2. SDP and RODP

Recently, many scholars have proposed a variety of defect prediction methods using some binary classification algorithms—e.g., neural networks [28], support vector machine [29], decision trees [30], Bayesian methods [31], and ensemble learning methods [32]. These proposed methods can learn from historical project datasets to construct a prediction model, and then predict whether or not the new module is defective (i.e., a binary classification task). However, only predicting the defect-proneness of the new software modules is not very efficient when the testing resource is very limited, because the CPDP model will allocate equal testing resource to all predicted defect-prone modules [33,34,35]. On the contrary, RODP can rank these new modules according to their predicted bug numbers or densities, thereby allocating the resources more efficiently.

In general, researchers use learning to rank techniques to construct the RODP models [18]. Learning to rank techniques can be divided into three categories, i.e., the pointwise method, the pairwise method, and the listwise method [36]. The pointwise method first uses some regression algorithm to predict the bug numbers of software modules, then uses these values to sort the modules. Recently, a lot of regression algorithms are employed to predict the bug numbers of software modules, including Poisson regression (PR) [37], genetic programming (GP) [38], decision tree regression (DTR) [39], etc. Moreover, Chen et al. [40] and Rathore et al. [41] conducted an empirical investigation of many regression algorithms to predict bug numbers, and the results showed that DTR, linear regression (LR), and Bayesian ridge regression (BRR) achieved better performance in terms of average absolute error (AAE) and root mean square error (RMSE). The pairwise method regard RODP problem as a binary classification task, i.e., constructing a classification model to identify which modules have more defects. For instance, Nguyen et al. [42] explored the performances of the pairwise Ranking SVM and RankBoost for RODP, and the results showed that the two algorithms outperformed the linear regression algorithm. The listwise method directly looks at the entire ranking list of modules by optimizing the performance measure. For instance, Yang et al. [18] proposed a RODP approach named LRT via directly optimizing the FPA metric.

### 2.3. CPDP

There is not sufficient training data for a new developed software project. However, there exist a lot of historical data from public software repositories [43]. To solve the insufficient training data problem, many CPDP methods are proposed. Zimmermann et al. [44] conducted an empirical study and found that if we directly used other project datasets as the training data, the built defect prediction model performed not well due to the different distribution between WP data and CP data. To alleviate the distribution gap, there exist the three main methods. The first method is to employ the training data selection methods to filter out irrelevant CP modules. For instance, Turhan et al. [19] proposed the nearest neighbor filter, and Peters et al. [21] proposed the Peters filter. Bin et al. [45] investigated nine training data selection methods for CPDP, and found that there seems to be no need to filter CP data. The second one is to use the transfer learning techniques to build the CPDP model. For instance, Ryu et al. [46] proposed the value-cognitive boosting with support vector machine method to transfer WP data information into the weights of the CP modules, and then build the predictor based on re-weighted CP modules. Jing et al. [16] employed the semi-supervised transfer component analysis (SSTCA) method to make the distributions of WP data and CP data consistent. The third method is to employ the unsupervised learning techniques that need not labeled training data sets for CCDP. For example, Zhang et al. [47] proposed to apply a spectral clustering algorithm to divide all WP modules into a defective cluster and a non-defective cluster.

## 3. Training Data Selection Method

In this section, we briefly introduce the training data selection methods.

### 3.1. Global Filter (GF): Using All CP Data

GF was proposed by Menzies et al. in [20]. GF employs all CP modules as the training data to construct the ROCPDP model, i.e., it does not filter out any CP modules.

### 3.2. Burak Filter (BF): WP Data Guided Filter

BF was proposed by Turhan et al. in [19]. It assumes that CP modules which are nearest to WP modules are the most valuable CP data. The detailed procedure of BF are as follows: (1) For each WP module, BF selects its *k* nearest neighbors in all CP modules based on the Euclidean distance. (2) BF combines these neighbors (without duplication) as the training data.

### 3.3. Peters Filter (PF): CP Data Guided Filter

PF was proposed by Peters et al. in [21]. It also assumes that CP modules that are nearest to WP modules are the most valuable CP modules. However, the details of PF are a little different from BF: (1) PF combines all WP modules and all CP modules, and uses the K-means algorithm to group these modules. (2) PF keeps the clusters containing at least one WP module. (3) For each CP module in each retained cluster, PF finds the nearest WP module in the same cluster. (4) After obtaining these WP modules, for each WP module, PF finds the neighbors from CP data in the same cluster. (5) PF combines these neighbors (without duplication) as the training data.

### 3.4. Kawata Filter (KF): Density-Based Spatial Clustering Guided Filter

KF was proposed by Kawata et al. in [22]. KF assumes that CP modules which are in the same cluster as WP modules are the most valuable modules in CP data. The details of KF are as follows: (1) KF combines all WP modules and all CP modules, and uses the DBSCAN algorithm to group these modules. (2) KF selects sub-clusters which consist at least one WP module, and collects the CP modules in the selected sub-clusters as the training data.

### 3.5. Yu Filter (YF): Hierarchical-Based Spatial Clustering Guided Filter

Our previous work proposed YF [23]. YF is similar to KF. The only difference is that YF uses the agglomerative clustering algorithm to group all WP modules and all CP modules, while KF uses the DBSCAN algorithm.

### 3.6. He Filter (HF): Distribution Characteristic Guided Filter

HF was proposed by He et al. in [24]. HF applies the distribution characters of every CP dataset to drive the selection process. HF selects training data at project level, while the aforementioned filters select training data at module level. HF selects k most similar CP datasets to the WP dataset, and combines all modules in the CP datasets as training data. The similarity is calculated as follows: (1) Each project dataset is represented as a distribution characteristic vector V = Min(f${}_{1}$), Max(f${}_{1}$), Median(f${}_{1}$), Mean (f${}_{1}$), STD(f${}_{1}$), …, Min(f${}_{n}$), Max(f${}_{n}$), Median(f${}_{n}$), Mean (f${}_{n}$), STD(f${}_{n}$), where Min(f${}_{i}$), Max(f${}_{i}$), Median(f${}_{i}$), Mean (f${}_{i}$) and STD(f${}_{i}$) represents the minimum value, the maximum value, the median value, the mean value, and the standard deviation value of the ith feature values of all modules in each project dataset. (2) The similarity of two project datasets is the Euclidean distance between the two distribution characteristic vectors of the two project datasets.

### 3.7. HeBurak Filter (HBF): Distribution Characteristic and WP Data Guided Filter

HBF was proposed by He et al. [24]. The details of HBF are as follows: (1) HBF applies HF to obtain the retrained CP datasets. (2) HBF applies BF to the retrained CP datasets to obtain the final training data.

### 3.8. HePerters Filter (HPF): Distribution Characteristic and CP Data Guided Filter

HPF was proposed by He et al. [24]. The details of HPF are as follows: (1) HPF applies HF to obtain the retrained CP datasets. (2) HPF applies PF to the retrained CP datasets to obtain the final training data.

### 3.9. Li filter (LF): Distribution Characteristic and Density-Based Partitioning Clustering Guided Filter

LF was proposed by Li et al. in [25]. The details of LF are as follows: (1) LF applies HF to obtain the retrained CP datasets. It is worth to note that LF uses the cosine distance to measure the similarity of projects, while original HF uses the Euclidean distance. (2) KF combines all WP modules and all CP modules in the retrained CP datasets, and uses the K-means algorithm to group these modules. (3) KF selects sub-clusters which consist at least one WP module, and collects the CP modules in the selected sub-clusters as the training data.

We adopt these training data selection methods in our experiments for the following reasons: Our selection of these training data selection methods closely resembles the choice by Bin et al. [45]. In addition, they investigated another two training data selection methods proposed in [20,48] for CPDP. The two methods train multiple individual defect prediction models to generate an ensemble CPDP model. To classify a new WP module, each individual model returns its class prediction, which counts as one vote. Then, the ensemble CPDP model counts the votes and assigns the class with the most votes to the new WP module. However, the two training data selection methods are not suitable for generating an ensemble ROCPDP model, because it is unfeasible to use the vote strategy to decide the rank of new modules. In addition, we set the parameters of the training data selection methods according to the original works [19,20,21,22,23,24,25].

## 4. Experimental Setup

### 4.1. Datasets

We used 11 widely used industrial project datasets from the PROMISE repository as our experimental datasets [49]. The detailed statistical results of the datasets are shown in Table 1, where #Module is the number of modules in the release, #Defects is the total number of defects in the release, %Defect is the percentage of defective-prone modules in the release, Max is the average value of defects of all defective-prone modules in the release, and Avg is the average value of defects of all defective-prone modules in the release. All datasets have the same 20 software features (metrics) [50,51,52] shown in Table 2.

### 4.2. Research Questions

**RQ1: Which training data selection method leads to better performance for ROCPDP?** For this question, following the settings used in previous studies [19], when we considered a project as a WP dataset, we chose other projects as the CP datasets. For instance, if we employed Ant 1.7 as the WP dataset, we used all other projects (i.e., Camel 1.6, Ivy 2.0, Jedit 4.3, Log4j 1.2, Lucene2.4, Poi 3.0, Synapse 1.2, Velocity 1.6, Xalan 2.7, Xerces 1.4) as the CP datasets. To deal with the randomness in the training data selection methods and prediction model, we ran the above procedure 100 times.

**RQ2: How does using training data selection methods and ROCPDP models perform compared to ROWPDP models trained on sufficient historical WP data?**

For this question, we wanted to investigate whether using training data selection methods and ROCPDP models can achieve performances comparable with those of ROWPDP models trained on sufficient historical WP data. Following the settings used in previous studies [19], for each project, the testing sets were selected from 10% of the data randomly. Defect prediction models were then constructed from:

- CP data: all data from the other projects.
- WP data: remaining 90% of the data of that project.

We ran the above procedure 100 times to avoid sample bias and deal with the randomness in the training data selection methods and prediction model.

**RQ3: How does using training data selection methods and ROCPDP models perform compared to ROWPDP models trained on limited WP data?**

For this question, we wanted to investigate whether using training data selection methods and ROCPDP models can achieve better performances than ROWPDP models trained on limited historical WP data. Following the settings used in previous studies, for each project, the testing sets are selected from 90% of the data randomly. Defect prediction models are then constructed from:

- CP data: all data from the other projects.
- WP data: remaining 10% data of that project.

We ran the above procedure 100 times to avoid sample bias and deal with the randomness in the training data selection methods and prediction model.

### 4.3. Performance Measures

In the experiment, we employed Norm(Popt) and FPA to evaluate the performances of SDP models, since the former measures the global ranking of modules according to the defect density, while the latter evaluates the global ranking of modules according to the number of defects. We explain Norm(Popt) and FPA as follows.

**Figure 2:**
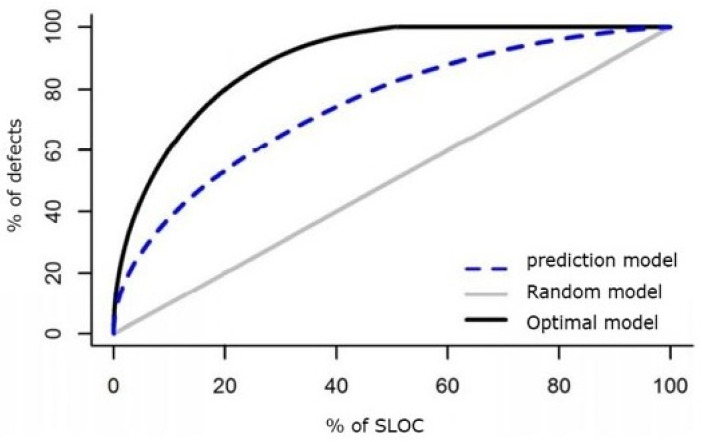
A SLOC-based cumulative lift chart.

In a the SLOC-based cumulative lift chart (Figure 2), the *x*-axis is the cumulative percentage of inspected SLOC, and the y-axis is the cumulative percentage of found defects.

(1) $$Norm\left(P_{\mathrm{opt}\;}\right)=\frac{P_{\mathrm{opt}\;}-\min\left(P_{\mathrm{opt}\;}\right)}{\max\left(P_{\mathrm{opt}\;}\right)-\min\left(P_{\mathrm{opt}\;}\right)}$$

Here, Popt is defined as 1-opt, where opt is the area between the optimal model and the prediction model in the SLOC-based cumulative lift chart. max(Popt) is the Popt value of the optimal model, while min(Popt) is the Popt value of the worst model (software modules are ranked by increasing actual defect densities).

FPA is the average of the proportions of actual defects in the top modules to the whole defects [18]. Assume that *n* modules in a project are ranked by no-decreasing order of the predicted bug numbers, as *M*${}_{1}$, *M*${}_{2}$, *M*${}_{3}$, …, *M*${}_{n}$, and *y* = *y*${}_{1}$ + *y*${}_{2}$ + …+ *y*${}_{n}$ is the total bug numbers of the *n* modules. Therefore, *M*${}_{n}$ is predicted to contain the most number of defects. The proportion of the actual bugs in the top m predicted modules to the whole bugs is calculated as:

(2) $$\frac{1}{y}\sum_{i=n-m+1}^{n}y_{i}.$$

Then, FPA is define as follows:

(3) $$FPA=\frac{1}{n}\sum_{m=1}^{n}\frac{1}{y}\sum_{i=n-m+1}^{n}y_{i}$$

The higher Norm(Popt) and FPA values indicate the better ranking, where the software modules that contain more defects or have higher defect density are ranked first.

### 4.4. Modeling Techniques

In this study, we used LTR [18] to build the ROWPDP and ROCPDP models. LTR is a listwise learning to rank approach, which trains a simple linear model:

(4) f(x)=〈w,x〉.

Then, it uses the composite differential evolution algorithm to directly optimize FPA to get *w*. Then, it employs the built model to predict the relative bug numbers or densities in new modules, and sort them according to the predicted values. Since parameter settings may impact the performances of ROCPDP models [53], we followed the original paper [18] to set the parameters of LTR; i.e., we set the feasible solution space to [−20, 20], and the population size and maximal generation were set to 100.

The reasons that we used LTR are as follows.

(1) Naïve Bayes (NB), logistic regression (LR), classification and regression tree (CART), bagging, random forest (RF), and k-nearest neighbors (KNN) have been widely used in SDP studies. These classification algorithms can rank all software modules with respect to their probability of containing defect(s). For example, the Naïve Bayes algorithm can output a score indicating the likelihood that a module is defective. However, these algorithms do not employ the information of the number of defects. In addition, Mende et al. [49] investigated the performances of these algorithms for RODP in terms of CE and Popt. Experimental results show that these algorithms had bad performances in terms of CE and Popt. Therefore, we do not employ these classification algorithms to build the ROCPDP model.
(2) The work that originally designed for RODP was limited. Yang et al. proposed LTR [18], which directly optimizes the performance measure (i.e., FPA) to obtain a ranking function. Chen et al. [40] and Rathore et al. [41] conducted an empirical investigation of many regression algorithms to predict bug numbers, and the results showed that DTR, LR, and BRR achieved better performance in terms of AAE and RMSE. Nguyen et al. [42] investigated Ranking SVM and RankBoost for RODP, and found that these algorithms outperformed the linear regression algorithm. Therefore, we compare LTR with DTR, LR, BRR, Ranking SVM and RankBoost. Experimental results showed that LTR significantly outperformed the compared algorithms in terms of FPA and Norm(Popt) using the 11 datasets in Table 1 through ten cross-validation. It is also consistent with the view of Liu et al. [36] that the listwise approach generally outperforms the pointwise approach and pairwise approach. Therefore, we use LTR to build the ROCPDP model.

### 4.5. Statistic Comparison Tests

(1) We computed the effect size, Cliff’s δ [54], to quantify the amount of difference between two methods. By convention, the magnitude of the difference is considered as trivial (|δ| < 0.147), small (0.147–0.33), moderate (0.33–0.474), or large (>0.474).
(2) Scott–Knott Test: Scott–Knott test [55] is a multiple comparison technique that employs hierarchical clustering algorithm to conduct the statistical analysis. The test divides the training data selection methods into significantly different groups. There is no significantly difference among the training data selection methods in the same group, whereas the training data selection methods in different groups have significant differences. In this study, we used the novel double Scott–Knott test [56] to cluster these training data selection methods into different groups: In the first step of the test, we divided the training data selection methods into significantly distinct groups with the 100 FPA and Norm(Popt) values on each dataset as the inputs. Therefore, each method had different rankings among different datasets. In the second step of the test, we used the Scott–Knott test to get the final rankings of the methods with all rankings of each method obtained in the first step as the input.

## 5. Experimental Results

In this section, we present the experimental results to answer the three research questions.

### 5.1. Which Training Data Selection Method Leads to Better Performance for ROCPDP?

**Figure 3:**
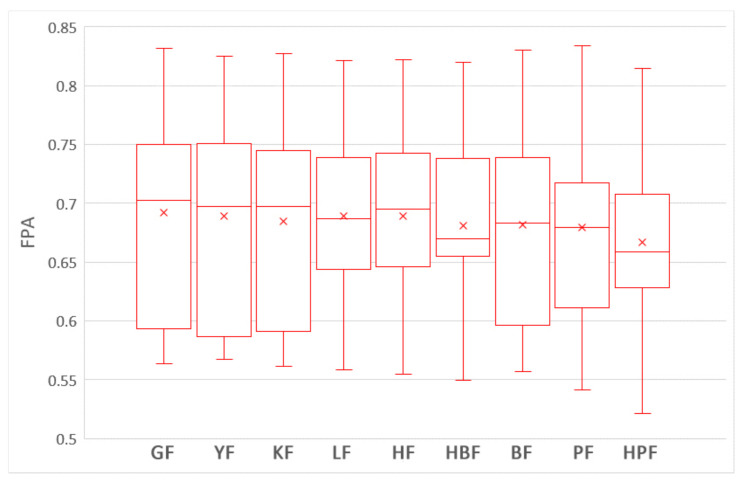
The boxplots of the nine training data selection methods with the Scott–Knott test results in terms of FPA.

**Figure 4:**
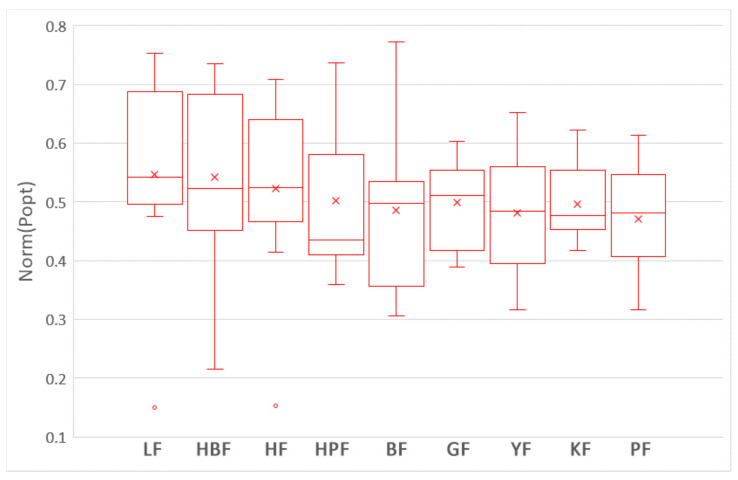
The boxplots of the nine training data selection methods with the Scott–Knott test results in terms of Norm(Popt).

To answer this question, we compare the impacts of nine training data selection methods on the performances of ROCPDP models. Following the visualization technique used in [47], the boxplots in Figure 3 and Figure 4 show the distributions of FPA and Norm(Popt) values of the methods with the Scott–Knott test results in the studied datasets. Different colors of the boxplot indicate different Scott–Knott test ranks (from top down, the order is red and blue). All methods in the same group are listed in decreasing order of the average FPA and Norm(Popt) values from left to right. From Figure 3 and Figure 4, we observe that the nine training data selection methods are clustered into a group in terms of FPA, which indicates that there exists no significant difference between these methods. In addition, the medium and average FPA values of GF are higher than those of the training data selection methods.

Table 3 shows the Cliff’s δ values of the nine training data selection methods in terms of FPA and Norm(Popt). White, light gray, deep gray, and yellow backgrounds indicate trivial, small, moderate, and large magnitudes of difference according to Cliff’s δ, respectively. As shown in Table 3, the differences among all methods are trivial or small in terms of FPA. In terms of Norm(Popt), the difference between LF and PF is large, the differences between LF and four other methods (GF, BF, KF, and YF) are moderate, the differences between HBF and two other methods (PF, and YF) are moderate, and the difference between HF and PF is moderate. In the other cases, the differences are trivial or small.

In summary, there are no significant differences among these nine training data selection methods in terms of FPA and Norm(Popt); and LF is moderately better than GF, BF, PF, KF, and YF in terms of Norm(Popt). The result is supported by the Scott–Knott test and Cliff’s δ effect size.

### 5.2. How Does Using Training Data Selection Methods and ROCPDP Models Perform Compared to ROWPDP Models Trained on Sufficient Historical WP Data?

**Figure 5:**
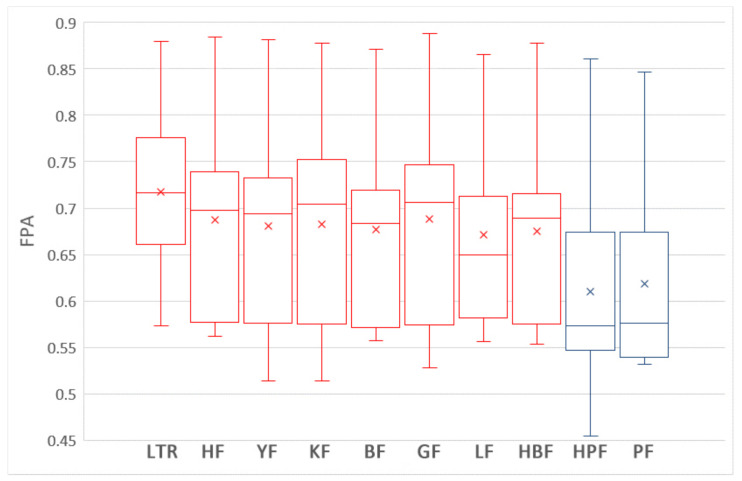
The boxplots nine training data selection methods and LTR trained on 90% WP data with the Scott–Knott test results in terms of FPA.

The boxplots in Figure 5 show the distributions of FPA values of the nine training data selection methods and LTR (i.e., ROWPDP model) trained on 90% WP data with the Scott–Knott test results. As shown in Figure 5, LTR, HF, YF, BF, GF, LF and HBF belong to the first group, while HPF and PF belong to the second group. In addition, the medium and average FPA values of LTR are higher than those of the training data selection methods.

**Figure 6:**
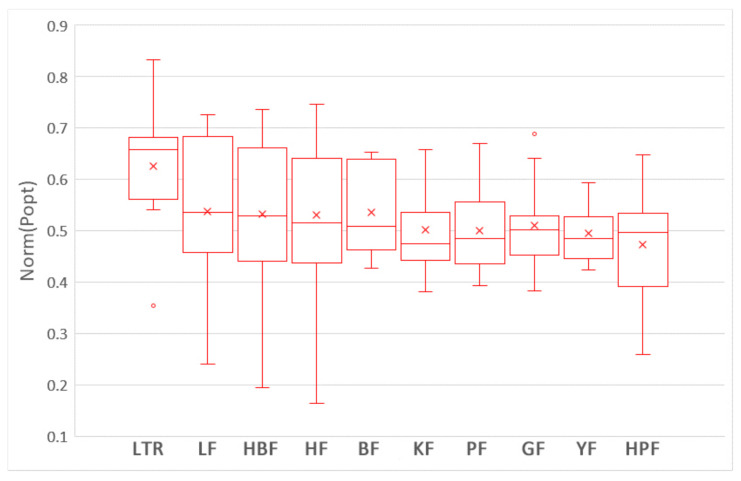
The boxplots of nine training data selection methods and LTR trained on 90% WP data with the Scott–Knott test results in terms of Norm(Popt).

The boxplots in Figure 6 show the distributions of Norm(Popt) values of the nine training data selection methods and LTR trained on 90% WP data with the Scott–Knott test results. As shown in Figure 6, we can observe that LTR and the nine training data selection methods are clustered into a group. In addition, the medium and maximum FPA values of LTR are much higher than those of the training data selection methods.

Table 4 shows the Cliff’s δ values of the nine training data selection methods and LTR trained on 90% WP data in terms of FPA and Norm(Popt). As shown in Table 4, the differences between LTR and two training data selection methods (PF and HPF) are large, and the differences between LTR and two training data selection methods (BF, HBF, and LF) are moderate in terms of FPA.

The differences between LTR and six training data selection methods (GF, BF, PF, KF, YF, and HPF) are large, and the differences between LTR and other three methods are moderate in terms of Norm(Popt). In summary, although Scott–Knott test results show that LTR and some training data selection methods belong to the same group, the Cliff’s δ effect size values indicate that there are large or moderate difference between LTR and most training data selection methods. In addition, LTR achieves the best average and median values in terms of FPA and Norm(Popt). Therefore, we can conclude that the performances of ROCPDP models trained on filter CP data using the training data selection methods are not comparable with those of ROWPDP models trained on sufficient historical WP data in terms of FPA and Norm(Popt).

### 5.3. How Does Using Training Data Selection Methods and ROCPDP Models Perform Compared to ROWPDP Models Trained on Limited WP Data?

**Figure 7:**
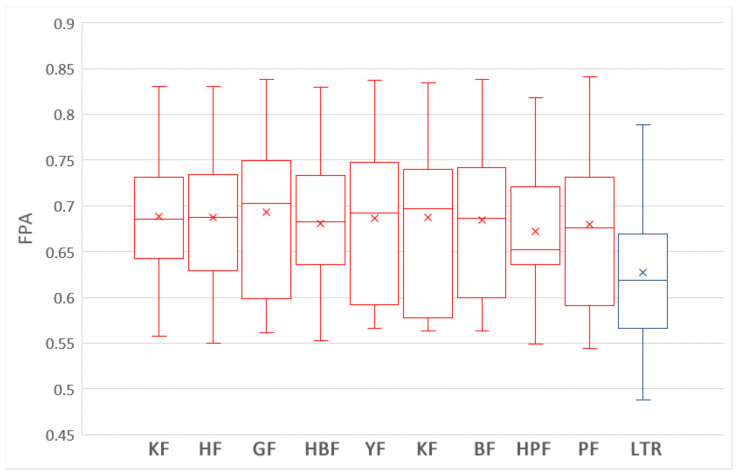
The boxplots of nine training data selection methods and LTR trained on 10% WP data with the Scott–Knott test results in terms of FPA.

**Figure 8:**
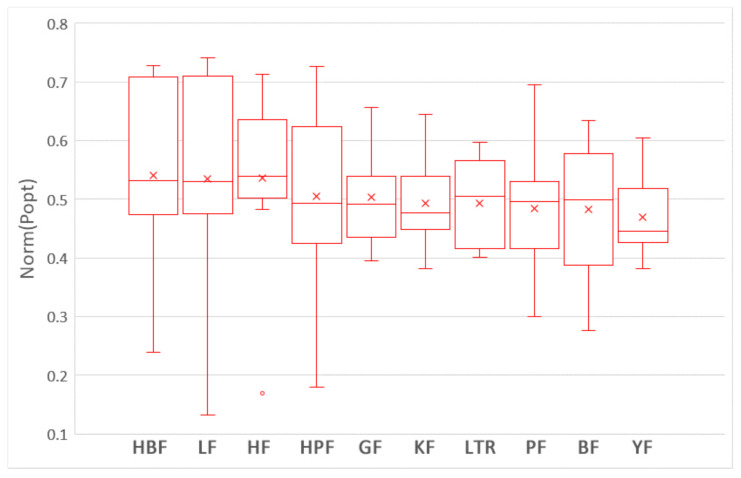
The boxplots of nine training data selection methods and LTR trained on 10% WP data with the Scott–Knott test results in terms of Norm(Popt).

The boxplots in Figure 7 show the distributions of FPA values of the nine training data selection methods and LTR (i.e., ROWPDP model) trained on 10% WP data with the Scott–Knott test results. As shown in Figure 7, all training data selection methods belong to the first group, while LTR belongs to the second group. It indicates that there are significant differences between the training data selection methods and LTR. In addition, LTR has the lowest average and medium FPA values. The boxplots in Figure 8 show the distributions of Norm(Popt) values of the nine training data selection methods and LTR trained on 10% WP data with the Scott–Knott test results. We can observe that the nine training data selection methods and LTR are clustered into a group. In addition, the average Norm(Popt) value of LTR is lower than those of six training data selection methods.

Table 5 shows the Cliff’s δ of the nine training data selection methods and LTR trained on sufficient WP data in terms of FPA and Norm(Popt). As shown in Table 5, the differences between LTR and all training data selection methods are moderate in terms of FPA, and the difference between LTR and two training data selection methods (GF and HBF) is small in terms of FPA and Norm(Popt). In summary, Scott–Knott test results and Cliff’s δ values show that there are significant differences between LTR and all training data selection methods in term of FPA. Although Scott–Knott test results show that LTR and all training data selection methods belong to the same group in terms of Norm(Popt), most training data selection methods achieve better average Norm(Popt) value than LTR. Therefore, we can conclude that the training data selection methods achieve better performance than ROWPDP models trained on limited historical WP data.

### 5.4. Discussion

We formulate and structure our experiment to address three research questions. The experimental results for RQ1 indicate that the data selection methods have no positive impacts on the performance of ROCPDP in terms of FPA and Norm(Popt). The experimental results for RQ2 show that the performances of the ROCPDP models trained on filter CP data are not comparable with those of ROWPDP models trained on sufficient historical WP data in terms of FPA and Norm(Popt). The experimental results for RQ3 indicate that ROCPDP models trained on filtered CP data achieved better performance values than ROWPDP models trained on limited historical WP data. Therefore, we suggest that software testers employ public project datasets to perform ROCPDP when there is no or limited WP data. In a similar study, Bin et al. [45] investigated the impacts of nine training data selection methods for CPDP, and also found that there is no need to filter CP data. The difference between Bin et al.’s study and ours is that we focused on ranking software modules according to the defect number or density, and we also investigated the impacts of training data selection methods on the performances of ROCPDP compared to ROWPDP models trained on sufficient and limited within-project data.

## 6. Conclusions and Future Work

In this study, we analyzed the performances of nine training data selection methods on ROCPDP models. We employed 11 industrial project datasets from the PROMISE repository as our experimental datasets, and used both a module-based effort-aware performance measure (FPA) and a SLOC-based effort-aware performance measure (Norm(Popt)) as the performance measures. We compared the ROCPDP models trained on the filtered cross-project data using the training data selection methods with the ROWPDP models trained on sufficient and limited within-project data. The experimental results showed that there was no significant difference among the nine training data selection methods. Although the performances of ROCPDP models with the filtered datasets cannot be comparable with those of ROWPDP models trained on sufficient historical WP data, they significantly outperformed ROWPDP models trained on limited historical WP data. Therefore, we recommend that software testers exploit other project datasets to perform ROCPDP when there are no or limited WP data. In our experiments, we employed 11 widely used industrial project datasets. For future studies, we plan to employ more project datasets to validate the generalization of our finding. Since the experimental results indicate that the existing data selection methods have no positive impacts on the performance of ROCPDP, developing a more effective training data selection method for ROCPDP is also one of our future research interests. In addition, we plan to design high-performing transfer learning algorithms to conduct ROCPDP. 

## Figures and Tables

**Table 1:** Details of Experiment Datasets.

Project	Description	#Modules	#Defects	%Defects	Max	Avg
Ant 1.7	A Java-based, shell independent build tool	125	33	16.0	3	1.65
Camel 1.6	An integration framework based on Enterprise Integration Patterns	965	500	19.5	28	2.66
Ivy 2.0	A dependence manager focusing on flexibility and simplicity	352	56	11.4	3	1.4
Jedit 4.3	A cross platform programmer’s text editor	492	12	2.2	2	1.09
Log4j 1.2	A logging package for printing log output	205	498	92.2	10	2.63
Lucene 2.4	A core search library	340	632	59.7	30	3.11
Poi 3.0	Java API for Microsoft documents format	442	500	63.6	19	1.78
Synapse 1.2	A lightweight and high-performance Enterprise Service Bus	256	145	33.6	9	1.69
Velocity 1.6	A template language engine	229	190	34.1	12	2.44
Xalan 2.7	An XSLT processor for transforming XML documents into HTML, text, or other XML document types	803	531	48.2	9	1.37
Xerces 1.4	A Java-based XML parser	588	1596	74.3	62	3.65

**Table 2:** The features of the datasets.

No.	Feature	Name	Description
1	wmc	Weighted methods per class	A class with more member functions than its peers is considered to be more complex and therefore more error prone
2	dit	Depth of inheritance tree	It’s defined as the maximum length from the node to the root of the tree
3	noc	Number of children	Number of direct descendants (subclasses) for each class
4	cbo	Coupling between object classes	Increased when the methods of one class access services of another
5	rfc	Response for a class	Number of methods invoked in response to a message to the object
6	lcom	Lack of cohesion in methods	Number of pairs of methods that do not share a reference to an instance variable
7	ca	Afferent couplings	How many other classes use the specific class
8	ce	Efferent couplings	How many other classes is used by the specific class
9	npm	Number of public methods	npm metric simply counts all the methods in a class that are declared as public
10	lcom3	Another lack of cohesion measure	*m*, *a* count the methods, attributes in a class. µ(*a*) is the number of methods accessing an attribute. $lcom3=\left(\left(\frac{1}{a}\sum_{j}^{a}\mu \left(a_{j}\right)\right)-m\right)/\left(1-m\right)$
11	loc	Lines of code	Total lines of code in this file or package
12	dam	Data access metric	Ratio of private (protected) attributes to total attributes
13	moa	Measure of aggregation	Count of the number of data declarations (class fields) whose types are user defined classes
14	mfa	Measure of functional abstraction	Number of methods inherited by a class plus number of methods accessible by member methods of the class
15	cam	Cohesion among methods of class	#different method parameters types divided by (#different method parameter types in a class)*(#methods)
16	ic	Inheritance coupling	Number of parent classes to which a given class is coupled (includes counts of methods and variables inherited)
17	cbm	Coupling between methods	Total number of new/redefined methods to which all the inherited methods are coupled
18	amc	Average method complexity	Number of Java byte codes
19	max_cc	Maximum McCabe’s cyclomatic complexity	Maximum McCabe’s cyclomatic complexity seen in class
20	avg_cc	Average McCabe’s cyclomatic complexity	Average McCabe’s cyclomatic complexity seen in class

**Table 3:** Cliff’s δ of the Nine Training Data Selection Methods in Terms of FPA and $Norm(P_{opt})$.

		$\mathrm{Norm}(P_{\mathrm{opt}})$								
		GF	BF	PF	KF	YF	HF	HBF	HPF	LF
FPA	GF		0.181	0.208	0.069	0.167	−0.236	−0.278	0.056	−0.389
	BF	0.180		0.083	−0.028	0.042	−0.292	−0.306	−0.069	−0.417
	PF	0.125	0.027		−0.167	−0.056	−0.375	−0.375	−0.069	−0.523
	KF	0.097	−0.125	−0.069		0.083	−0.292	−0.306	0.083	−0.458
	YF	0.027	−0.152	−0.097	−0.042		−0.319	−0.347	−0.042	−0.431
	HF	0.069	−0.111	−0.097	0.042	0.042		−0.111	0.194	−0.167
	HBF	0.194	0.083	0.138	0.125	0.166	0.208		0.222	−0.097
	HPF	0.278	0.194	0.166	0.208	0.236	0.277	0.181		−0.319
	LF	0.097	−0.125	−0.111	0	0.111	0.083	−0.125	−0.292	

**Table 4:** Cliff’s δ of the Nine Training Data Selection Methods and LTR Trained on 90% WP Data in Terms of FPA and $\mathrm{Norm}(P_{opt})$.

		$\mathrm{Norm}(P_{\mathrm{opt}})$									
		GF	BF	PF	KF	YF	HF	HBF	HPF	LF	LTR
FPA	GF		−0.181	0.042	0.069	0.194	−0.25	−0.306	0.125	−0.194	−0.638
	BF	0.153		0.25	0.264	0.333	−0.027	−0.083	0.333	−0.042	−0.569
	PF	0.402	0.431		0.014	0.014	−0.236	−0.319	0.125	−0.263	−0.708
	KF	0.056	−0.111	−0.389		0.014	−0.194	−0.264	0.139	−0.236	−0.694
	YF	0.083	−0.097	−0.403	0.014		−0.333	−0.333	0.042	−0.264	−0.75
	HF	0.055	−0.125	−0.486	−0.014	−0.069		−0.056	0.264	−0.014	−0.444
	HBF	0.111	0	−0.403	0.056	0.056	0.514		0.319	−0.056	−0.444
	HPF	0.458	0.472	−0.414	0.458	0.458	0.153	0.444		−0.333	−0.75
	LF	0.153	0.097	−0.403	0.097	0.153	0.153	0.056	−0.389		0.361
	LTR	−0.194	−0.361	−0.597	−0.25	−0.292	−0.222	−0.333	−0.667	−0.389	

**Table 5:** Cliff’s δ of the Nine Training Data Selection Methods and LTR Trained on 10% WP Data in Terms of FPA and $Norm(P_{opt})$.

		$\mathrm{Norm}(P_{\mathrm{opt}})$									
		GF	BF	PF	KF	YF	HF	HBF	HPF	LF	LTR
FPA	GF		0.069	0.097	0.111	0.278	−0.375	0.306	−0.028	−0.292	0.278
	BF	0.056		0.014	0.042	0.194	−0.361	−0.306	−0.069	−0.278	0.014
	PF	0.125	0.056		0.069	0.194	−0.431	−0.375	−0.083	−0.347	−0.083
	KF	0.056	−0.028	−0.097		−0.111	−0.042	−0.069	0.222	−0.472	0.056
	YF	0.069	−0.056	−0.056	−0.014		−0.611	−0.486	−0.208	−0.528	−0.032
	HF	0.111	−0.042	−0.083	0.069	0.014		−0.056	0.25	0.056	0.111
	HBF	0.166	0.097	−0.042	0.139	0.125	0.097		0.236	0.042	0.166
	HPF	0.236	0.208	0.083	0.236	0.222	0.222	−0.139		−0.236	0.027
	LF	0.111	0	−0.083	0.506	0.027	−0.028	−0.097	−0.25		0.125
	LTR	0.416	0.389	0.375	0.403	0.403	0.458	0.431	0.347	0.444	

## Data Availability

Not applicable.
